# Endometrial sampling at an academic hospital in South Africa: Histological findings, lessons learnt and interesting surprises

**DOI:** 10.4102/ajlm.v9i1.1038

**Published:** 2020-09-29

**Authors:** Reena D. Mohanlal

**Affiliations:** 1 Department of Anatomical Pathology, Faculty of Health Sciences, University of the Witwatersrand, Johannesburg, South Africa; 2 National Health Laboratory Services, Chris Hani Baragwanath Laboratory, Johannesburg, South Africa

**Keywords:** Endometrial sampling, inadequate endometrial biopsy, postmenopausal bleeding, pathology, histopathology, gynaecology

## Abstract

**Background:**

Outpatient sampling is used to investigate endometrial pathology. Little is known about practice habits and local failure rates at Chris Hani Baragwanath Academic Hospital in Johannesburg, South Africa.

**Objective:**

This study assessed the frequency of samples that showed no or limited histological representation of endometrium, and described demographic and pathological features.

**Methods:**

All endometrial sample histology reports from the National Health Laboratory Services at the hospital from 01 July 2013 to 31 May 2017 were retrieved by searching the laboratory’s information system. Clinical variables (age, menopausal state, indication for biopsy, endometrial thickness on ultrasound) and pathological findings (macroscopic amount of tissue, histological diagnosis, microscopic presence of endometrial tissue) were extracted and statistically analysed.

**Results:**

A total of 1926 samples were included, 91% of which were submitted for abnormal or postmenopausal bleeding. No endometrium was observed in 25% of samples and 13% showed limited endometrium. Benign diagnoses (86%) were most common, with proliferative or secretory changes, endometrial polyps and endometritis accounting for most of these. Associations between the amount of sample received and the presence of endometrial tissue (*p* ≤ 0.001) and benign versus malignant diagnoses (*p* ≤ 0.001) were noted. The greater the endometrial thickness, the greater the likelihood of obtaining more sample (bulky vs scant *p* < 0.001) and making a malignant versus benign diagnosis (*p* = 0.005).

**Conclusion:**

These findings are in keeping with literature outside Africa. Histology reports should be explicit when terms such as ‘inadequate’ or ‘insufficient’ are used, in order to facilitate clinical decision-making.

## Introduction

Outpatient blind endometrial sampling using a disposable, flexible aspiration device such as a Z-sampler or pipelle is well tolerated by patients and is a quick and easy procedure to perform.^1^ In resource-limited settings, this modality for investigating suspected endometrial disease becomes especially relevant, as it eliminates the cost of a hospital stay, anaesthetic administration and post-operative complications inherent with in-theatre diagnostic dilation and curettage. Most of the literature pertaining to endometrial sampling is from non-African countries. Outpatient sampling compares well to other modalities and studies have shown a concordance rate of 95% between pipelle and diagnostic dilation and curettage for atypia and hyperplasia,^1^ agreement between office sampling and final diagnosis of 0.73, and no significant difference between hysteroscopic and office sampling.^2^ Outpatient sampling has also been shown to avoid hysteroscopy, particularly in patients with postmenopausal bleeding and an endometrial thickness of more than 4 mm.^3^ There were no significant differences in the agreement rates for tumour type or grading among endometrial biopsy, curettage or hysteroscopy methods.^4^ However, outpatient sampling performs less well when there are focal endometrial lesions.^1^ The biopsy procedure may fail when insufficient tissue is obtained or there is an inability to access the endometrial cavity.^5^ Failure rates of up to 53% have been cited in a meta-analysis.^5^ Furthermore, the reporting of these biopsies is not standardised. There is poor agreement on what constitutes an ‘insufficient’ or ‘inadequate’ biopsy^6,7^ among both general and gynaecologic pathologists, as there are no standardised criteria for determining adequacy as shown in a study from the United Kingdom.^7^ Such ambiguous sign-outs or reporting may result in misinterpretation by the clinician and subsequent overtreatment of the patient.^6^ This study was undertaken to determine the frequency of biopsies of outpatient endometrial aspiration that showed no or limited amounts of endometrium and to describe demographic and pathological findings in a retrospective cohort of cases at a tertiary hospital in Johannesburg, South Africa.

## Methods

### Ethical considerations

Ethics approval for this study was obtained from the Medical Ethics Committee of the University of the Witwatersrand (M170697).

### Study design

This retrospective, descriptive cross-sectional study was conducted at the Department of Anatomical Pathology, Chris Hani Baragwanath Hospital National Health Laboratory Services, located on the hospital premises in Soweto, South Africa. Endometrial biopsies are submitted to the laboratory from the gynaecology department at the hospital. A retrospective search of all endometrial biopsies reported from 01 July 2013 to 31 May 2017 was conducted using the search word ‘endometrium’ on the laboratory’s information system. A review of all histology reports identified using this search was then undertaken.

All endometrial biopsy samples obtained with an aspiration device such as a Z-sampler were included; while those biopsy samples obtained during hysteroscopy, dilatation and curettage and suction curettage were excluded. Clinical and pathological variables were obtained from the histology reports only. No clinical records or patients’ files were accessed. Age, menopausal status, endometrial thickness (in millimetres), clinical examination findings, the presence of risk factors for carcinoma (body mass index ≥ 25 kg/m^2^, hypertension, diabetes, tamoxifen use, nulliparity) and indications for biopsy were obtained from the clinical history section of the histopathology reports. The clinical history section on the histology report comprises information provided by the submitting clinician on the requisition slip that accompanies a specimen. Pertinent laboratory findings including diagnosis, diagnostic category (benign, atypical, malignant), quantity of tissue at macroscopic assessment (scanty, moderate or bulky) and the presence or absence of endometrial tissue on microscopic examination were also recorded from the histology report.

**FIGURE 1:**
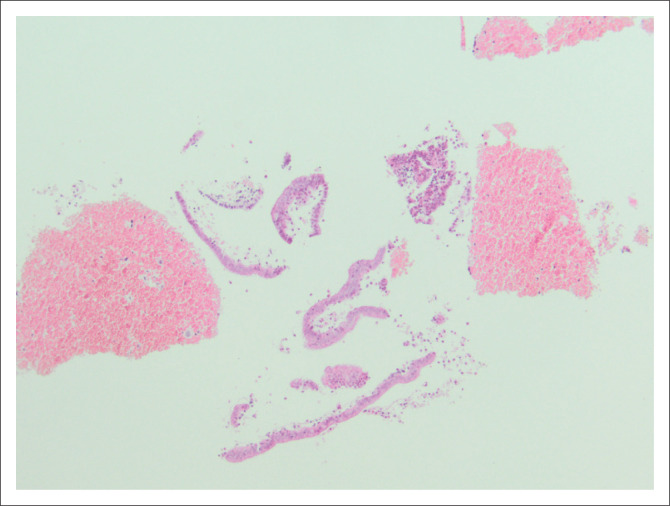
Endometrial sample showing limited endometrial tissue comprising less than 10 endometrial strips (original magnification ×100).

Cases with superficial strips of endometrium or stroma recorded on histological examination were classified as limited (Figure 1). Diagnoses recorded as malignant were carcinomas and lymphomas; those recorded as atypical were hyperplasia with atypia and squamous intraepithelial lesions. All remaining cases, for example polyps, endometritis, and proliferative or secretory phase endometrium, were categorised as benign. As per laboratory standard operating procedures, the categories of tissue assigned at grossing, or macroscopic examination, were: bulky tissue (fills more than one tissue cassette), moderate (fills up to one tissue cassette) and scanty (minimal or small fragments of tissue submitted). Slides of selected cases were retrieved for photography.

### Data analysis

All data were recorded on a datasheet and entered into an Excel (Microsoft, Redmond, Washington, United States) spreadsheet for statistical analysis. Where data were missing, no value was entered. There was no patient follow-up or clinical record review. Statistica^TM^ version 13.0 (Dell, Round Rock, Texas, United States) and STATA version 15 (Statacorp, College Station, Texas, United States) were used in the statistical analysis. Categorical data (macroscopic tissue quantity, diagnostic category, diagnosis) were reported as frequencies. Numerical data (age and endometrial thickness) were reported as medians with interquartile ranges, as these data were non-normally distributed. Only endometrial thicknesses reported in postmenopausal patients were used. The chi-square and Fisher’s exact tests were used to assess associations between the amount of tissue at grossing and diagnostic category, and the presence of endometrial tissue on histological examination. The Kruskall–Wallis test was used to assess differences in endometrial thicknesses recorded for macroscopic quantity of tissue and diagnostic category. Pairwise comparison (Dunn’s test) was used to assess associations between median endometrial thickness and diagnostic category, and macroscopic tissue quantity in the postmenopausal group. Statistical significance was set at *p* < 0.05.

## Results

### Entire cohort

A total of 1926 outpatient endometrial aspiration biopsies were received and reported in the laboratory from 01 July 2013 to 31 May 2017. The median age of the patients was 53 (interquartile range 48–62) years (Table 1). Most biopsies (*n* = 1625, 90.58%) were submitted for abnormal or postmenopausal bleeding. In 196 (10.18%) cases, the presence of risk factors for endometrial carcinoma was indicated (data not shown in table). Scanty and moderate samples comprised the majority of samples received, with only 135 (6.82%) bulky specimens noted. For the entire group, 485 (25.18%) cases had no endometrium and 245 (12.72%) cases had limited amounts in the form of superficial strips of mucosa or stroma (Figure 1). The majority of diagnoses were benign (*n* = 1725, 86.42%). Of the 110 malignancies diagnosed, there were 32 cases of serous carcinoma of the endometrium, 24 endometrioid carcinomas and 13 malignant mixed Müllerian tumours. Cervical pathology was noted in 107 (5.56%) of the total biopsies, 70 cases of which were diagnosed with cervical squamous intraepithelial lesions, eight with squamous cell carcinoma and three with cervical adenocarcinoma.

**TABLE 1:** Clinical characteristics and pathological findings for all endometrial samples received at Chris Hani Baragwanath Academic Histopathology Laboratory, South Africa, 01 July 2013 – 31 May 2017.

Variable	*n*	%	IQR
**Age†**			
53 years	-	-	48–62
**Indications**			
Postmenopausal bleeding	964	53.73	-
Abnormal uterine bleeding	661	36.85	-
Per vaginal discharge	12	0.67	-
Post-coital bleeding	5	0.28	-
Abnormal Pap smear	24	1.34	-
Lower abdominal pain	30	1.67	-
Mass	14	0.78	-
Increased endometrial thickness	26	1.45	-
Work-up for malignancy	15	0.84	-
Miscellaneous	113	6.3	-
Missing	132	-	-
**Menopausal status**			
Perimenopausal	68	3.53	-
Reproductive years	91	4.72	-
Postmenopausal	994	51.61	-
Not indicated	773	40.13	-
**Macroscopic quantity**			
Scanty	827	41.77	-
Moderate	935	47.22	-
Bulky	135	6.82	-
Missing	29	-	-
**Diagnostic category**			
Benign	1725	86.42	-
Malignant	110	5.71	-
Atypical	91	4.72	-
**Endometrial tissue**			
Present	1196	62.10	-
Absent	485	25.18	-
Limited	245	12.72	-
**Diagnoses§**			
Proliferative phase endometrium	298	-	-
Endometrial polyp	194	-	-
Endometritis	189	-	-
Inactive endometrium	182	-	-
Secretory phase endometrium	94	-	-
Atrophic endometrium	26	-	-
Actinomyces	6	-	-
Endometrial hyperplasia	21	-	-
Endometrial hyperplasia with atypia	18	-	-
Endometrial malignancies			
Endometrial carcinoma	96	-	-
Serous carcinoma	32	-	-
Endometrioid carcinoma	24	-	-
Malignant mixed Müllerian tumour	13	-	-
Endometrial neuroendocrine carcinoma	3	-	-
Carcinoma not otherwise specified	23	-	-
Clear cell carcinoma	1	-	-
Non-Hodgkin lymphoma	1	-	-
Ovarian serous carcinoma	1	-	-
Cervical pathology			
Endocervical polyp	20	-	-
Cervical squamous intraepithelial lesion	70	-	-
Squamous cell carcinoma	8	-	-
Cervical adenocarcinoma	3	-	-
Neuroendocrine carcinoma of the cervix	1	-	-
Endocervicitis	5	-	-

IQR, interquartile range.

† , Age is expressed as median and interquartile range in parentheses.

§ , The total number of samples was 1926. In some cases, there were multiple diagnoses or no diagnosis.

The distribution of samples with histologically determined endometrium present, absent or limited in amount and the diagnostic categories across macroscopic quantities was assessed. Associations between the macroscopic quantity of tissue received and the presence of endometrial tissue (*p* ≤ 0.001) and diagnostic category (*p* ≤ 0.001) are shown in Table 2.

**TABLE 2:** Macroscopic quantities of endometrial tissue samples received at Chris Hani Baragwanath Academic Hospital Histopathology Laboratory from 01 July 2013 to 31 May 2017 in relation to microscopically assessed endometrial tissue and diagnostic categories.

Variable	Scanty (*n* = 827)		Moderate (*n* = 935)		Bulky (*n* = 135)		*p*
	*n*	%	*n*	%	*n*	%	
**Histologically assessed endometrium**							< 0.001*
Endometrium present	292	34.46	766	81.93	122	90.37	
No endometrium	355	42.93	112	11.98	7	5.19	
Limited endometrium	180	21.77	57	6.10	6	4.44	
**Diagnostic category as per histological findings**							< 0.001**
Benign	771	93.23	820	87.70	106	78.52	
Malignant	19	2.30	68	7.27	22	16.30	
Atypical	37	4.47	47	5.03	7	5.19	

Note: The cases used were all those where macroscopic tissue quantity was stated in the report, *n* = 1897.

* , Chi-square tests was used.

** , Fisher’s exact test was used.

### Cases of interest

**FIGURE 2:**
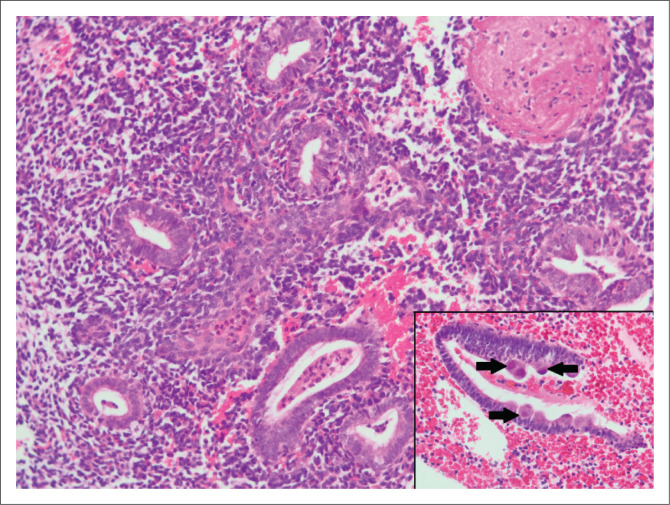
Endometrial breakdown and endometritis in an endometrial sample from a 39-year-old patient with abnormal bleeding (original magnification ×200). Cytomegalovirus inclusions in endometrial epithelial cells are indicated by arrows in the inset (original magnification ×400).

**FIGURE 3:**
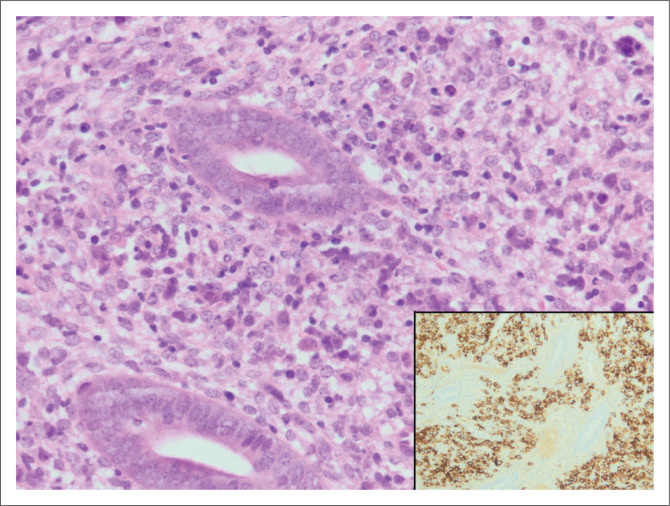
Diffuse large B-cell non-Hodgkin lymphoma infiltrating endometrial stroma in a sample from a 22-year-old patient with lymphadenopathy and organomegaly (original magnification ×400). CD20 immunohistochemical stain showing CD20-positive B-cells stained brown (inset, original magnification ×200).

Two cases of interest were noted. An endometrial sample from a 39-year-old woman with abnormal uterine bleeding revealed cytomegalovirus endometritis (Figure 2). No clinical follow-up was available for this patient. A B-cell non-Hodgkin lymphoma (Figure 3) was noted in a 22-year-old woman with bilateral ovarian masses, hepatosplenomegaly and lymphadenopathy. In addition to endometrial involvement, previous histology also showed nodal and marrow infiltration by lymphoma.

### Actinomyces

**FIGURE 4:**
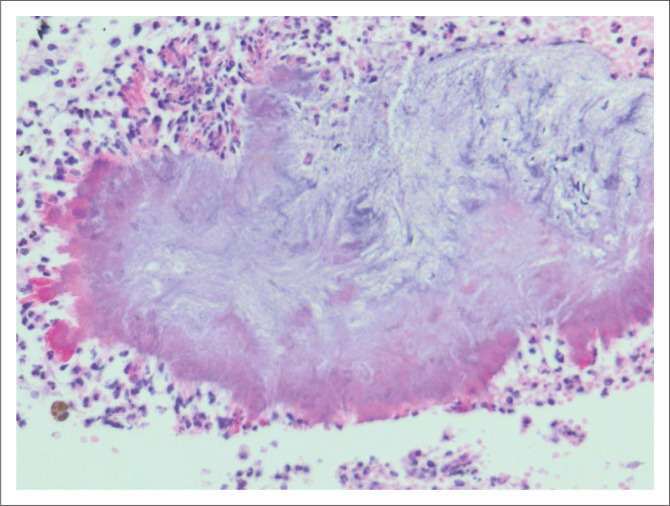
*Actinomyces* bacterial infection in an endometrial sample (original magnification ×400) from a 57-year-old female who presented with postmenopausal bleeding. The bacteria are seen as the large clumped mass of basophilic filamentous structures.

Six biopsies from five patients, ranging in age from 54 to 78 years, showed infection with *Actinomyces* bacteria (Figure 4). All patients with *Actinomyces* infection presented with abnormal bleeding, with one patient additionally complaining of per vaginal discharge. An intrauterine contraceptive device in situ was noted in only one patient and none of patients with *Actinomyces* infection had microbiological confirmation.

### Tamoxifen use

Of the total biopsies studied, 11 were from patients on tamoxifen. Three presented with postmenopausal bleeding, and the endometrial thickness reported in three women ranged from 4 mm to 18 mm (data not shown). Two biopsies from patients on tamoxifen showed endometrial polyps (both patients presented with bleeding), two showed endometritis and one, atypical squamous cells (data not shown).

### Postmenopausal subgroup

A total of 994 patients (see Table 1) of the entire cohort were postmenopausal, as indicated in the clinical history sections of the laboratory records. The median age in the postmenopausal group was 60 years (interquartile range 54–68). The majority of biopsies (*n* = 964, 98.27%), where an indication for biopsy was provided, were for postmenopausal bleeding. Risk factors for malignancy were noted in 126 (12.7%) patients (data not shown). In the postmenopausal group, 267 (26.86%) had no endometrium and 152 (15.29%) had limited amounts (data not shown). Malignant diagnoses accounted for 82 (8.27%) of postmenopausal cases (data not shown). Other significant pathological findings included hyperplasia (*n* = 16), hyperplasia with atypia (*n* = 15), endometrial polyps (*n* = 115), inactive endometrium (*n* = 125) and endometritis (*n* = 71) (data not shown). Endometrial thicknesses were specified in only 94 of postmenopausal patients, with an overall median of thickness of 11.0 mm (interquartile range 7–17) (data not shown).

Significant differences were noted in the median endometrial thicknesses recorded among the diagnostic categories and macroscopic quantities of tissue for postmenopausal women (Table 3). On subsequent analysis, it was shown that the greater the endometrial thickness, the greater the likelihood of obtaining more tissue (bulky vs moderate *p* = 0.021 and bulky vs scanty *p* < 0.001) (data not shown). In addition, the thicker the endometrium measured on ultrasound, the more likely a diagnosis of malignant versus atypical (*p* = 0.048) and malignant versus benign (*p* = 0.005) (data not shown) was made.

**TABLE 3:** Median endometrial thicknesses per diagnostic category and macroscopic quantity of tissue in postmenopausal women obtained by endometrial sample at Chris Hani Baragwanath Academic Hospital, South Africa, 01 July 2013 – 31 May 2017.

Variable	*n*†	Median endometrial thickness (IQR) in mm‡		p*
		mm	IQR	
**Diagnostic category**				0.0324
Benign	81	10	6–16	
Atypical	4	11	7.25–13.25	
Malignant	9	18	13.5–26	
**Macroscopic tissue quantity**				0.0011
Bulky	11	19	15–21	
Moderate	43	12	9–17	
Scanty	40	8	5–13	

IQR, interquartile range; *n*, absolute number.

* , *p*-values obtained using the Kruskall–Wallis test.

† , Total number of patients = 94.

‡ , Cases include those where postmenopausal status and endometrial thickness was provided by the clinician on the accompanying requisition slip.

## Discussion

Outpatient endometrial samples are regularly encountered in general pathology practice. One-quarter of samples in this study contained no endometrial tissue on histological examination. Furthermore, in the postmenopausal group, endometrial thickness on ultrasound correlated with the amount of tissue obtained at sampling and the histologically confirmed diagnostic category.

As per other studies from Canada and Turkey, abnormal bleeding was the most common presenting feature in our study.^8,9^ The importance of adhering to guidelines or indications for biopsy has been stressed, as endometrial sampling is not without risk. A tendency to over-investigate patients younger than 40 with abnormal bleeding has been shown.^10^ Furthermore, sampling of the endometrium without a clear indication may contribute to higher failure rates.

Although larger samples show better agreement rates with final excisional diagnoses,^4,11^ bulky specimens formed the minority of samples submitted in our cohort. Failure rates reported in the literature include cases where insufficient tissue was noted on histological examination or where the endometrium could not be accessed due to technical or patient-related factors.^5^ Higher failure rates have been noted in postmenopausal, obese patients, those with advanced age or previous failed pipelle and when sampling was performed by a non-physician.^9^ The failure rate for this cohort is higher than some reported failure rates (6.33% – 18.4%)^8,9,12^ and lower than the overall failure rate of 42% reported in a meta-analysis where 11% of cases failed due to technical reasons and 31% had insufficient tissue.^5^ These figures need to be interpreted with caution, as the criteria for labelling biopsies as ‘insufficient’ may vary across institutions. In one study, complete agreement on classification as insufficient or diagnostic was shown in only 57% of cases.^7^ The terms ‘insufficient’ or ‘inadequate’ were proposed for cases with no endometrial tissue present and ‘unassessable’ if there was too little tissue.^6^ Recently published Canadian guidelines may facilitate standardising reporting. Categories of ‘non diagnostic sample, no endometrial tissue present’ and ‘scant fragments of inactive endometrial surface epithelium and/or stroma (suboptimal for histopathological assessment)’ have been suggested.^13^ An endometrial surface area of at least 35 mm,^2,11^ or a minimum of 10 endometrial strips^14^ have also been proposed as cut-offs for a conclusive sample. Limited endometrial tissue was noted in 245 (12.72%) of our biopsies. The histological finding of limited endometrial tissue should be correlated clinically with the endometrial thickness as this microscopic finding could be compatible with atrophy, requiring no further clinical intervention. Adequacy is related to endometrial thickness. One study showed that 27% of samples were adequate among postmenopausal patients with an endometrial thickness of less than 5 mm, leading to the suggestion that sampling should not be done in this group.^3^ This study corroborates the findings of others in that the thicker the endometrium in postmenopausal women, the greater the likelihood of obtaining more sample and a malignant diagnosis.^3,8^ Specificity of endometrial sampling is high, but its sensitivity is low.^5^ One study has shown that, following an inadequate or scant biopsy, 10% of patients had hyperplasia, 5% had malignancy on repeat sampling and 15% had malignancy on hysterectomy.^7^ Worrisome histological findings such as atypia or necrosis on initial biopsy should be communicated as 43% of such patients in one study had malignancy on follow-up biopsy.^15^

The frequency of histological diagnoses noted in the entire cohort is similar to findings by Inal et al.,^8^ with the exception that hyperplasia was diagnosed in fewer patients (2.02% vs their 9.7%). Cervical pathology was noted in some of this cohort’s endometrial biopsies. Histopathologists should be alert to the presence of, and pathological changes in, cervical tissue present in an endometrial sample, as this may direct clinicians to re-examine and rebiopsy the cervix. In the current study, hyperplasia or malignancy were diagnosed in 113 (11.37%) of postmenopausal women, which is in keeping with other studies.^10,16^

Cytomegalovirus endometritis and endometrial involvement by non-Hodgkin lymphoma were two interesting cases encountered. Isolated case reports of cytomegalovirus endometritis from the 1990s have been noted, but the most recent report was in a postpartum uterus specimen.^17^ Polymerase chain reaction for cytomegalovirus was negative and the diagnosis was based on immunohistochemistry.^17^ A caveat to bear in mind is that biotin present in postpartum endometrial epithelium interacts with the avidin-biotin-complex, resulting in false-positive staining on immunohistochemistry.^18^ In women, non-Hodgkin lymphoma most commonly affects the ovaries, as it is postulated that the ovarian microenvironment is conducive to the growth of tumour cells. It is important to categorise lymphomas as either primary genital tract disease or secondary involvement, as this may have treatment implications.^19^ The prevalence of actinomycotic endometritis was 0.31% in this study, which is higher than the 0.0002% previously recorded.^20^ The association with prolonged use of an intrauterine contraceptive device is well known, but this history is not always forthcoming. Although forming only a very small subset in this study, patients on tamoxifen with postmenopausal bleeding and with adequate endometrial tissue most commonly had endometrial polyps on biopsy, which is similar to another study.^21^

### Limitations

This study included a review of laboratory-record histology reports from a large number of endometrial biopsies. Clinical information submitted in some instances was minimal, with no information about the menopausal status or endometrial thickness. The postmenopausal group was determined using clinical history only and all cases with no comment on menopausal state were excluded from this subgroup. A large number of cases could therefore have been left out, but assumptions about the menopausal state based purely on age could not be justified. Furthermore, there was no clinical record review to assess outcomes and final diagnosis for cases where there was no endometrium or limited endometrium. As this hospital is a tertiary public hospital in a metropolitan area, the cases are biased and therefore may not be a true reflection of the South African community. Further areas of study could include a follow-up of cases with no or limited endometrium and to assess local reasons for the failure rates in this study.

### Conclusion

This study provides information about the indications, diagnoses and representation of endometrium in endometrial samples seen at a large tertiary hospital in Soweto. There should be an awareness among pathologists and clinicians regarding terms such as ‘insufficient’, ‘inadequate’, ‘scant’ and ‘unassessable’ in an endometrial sample pathology report. If these terms are used, they should be accompanied by a clear description of the type and amount of tissue that is present as there is no consensus among pathologists regarding adequacy criteria.^6,7^ In the appropriate setting, repeat biopsy can be suggested by the pathologist when no or little endometrial tissue is present as there is a possibility of finding hyperplasia or malignancy on subsequent biopsy.^7^ Ultimately, management following an ‘insufficient’ tissue biopsy will depend on clinical factors such as the persistence of bleeding and ultrasound findings.^7,12,13^ Endometrial sampling is a useful investigation if done under the appropriate clinical circumstances, submitted with adequate history and reported mindfully by the histopathologist.
